# Machine learning models to predict success of endoscopic sleeve gastroplasty using total and excess weight loss percent achievement: a multicentre study

**DOI:** 10.1007/s00464-023-10520-0

**Published:** 2023-11-16

**Authors:** Maria Vannucci, Patrick Niyishaka, Toby Collins, María Rita Rodríguez-Luna, Pietro Mascagni, Alexandre Hostettler, Jacques Marescaux, Silvana Perretta

**Affiliations:** 1 General Surgery Department, University of Torino, Turin, Italy; 2 Research Institute Against Digestive Cancer (IRCAD), Strasbourg, France; 3 Research Institute Against Digestive Cancer (IRCAD), Kigali, Rwanda; 4 ICube Laboratory, Photonics Instrumentation for Health, Strasbourg, France; 5 Fondazione Policlinico Universitario Agostino Gemelli IRCCS, Rome, Italy; 6 Research Group CAMMA, University of Strasbourg, Strasbourg, France; 7 Department of Digestive and Endocrine Surgery, University of Strasbourg, Strasbourg, France; 8 IHU-Strasbourg, Institute of Image-Guided Surgery, Strasbourg, France; 9 Turin, Italy

**Keywords:** Bariatric endoscopy, Machine learning, Predictive model, Interventional endoscopy

## Abstract

**Background:**

The large amount of heterogeneous data collected in surgical/endoscopic practice calls for data-driven approaches as machine learning (ML) models. The aim of this study was to develop ML models to predict endoscopic sleeve gastroplasty (ESG) efficacy at 12 months defined by total weight loss (TWL) % and excess weight loss (EWL) % achievement. Multicentre data were used to enhance generalizability: evaluate consistency among different center of ESG practice and assess reproducibility of the models and possible clinical application. Models were designed to be dynamic and integrate follow-up clinical data into more accurate predictions, possibly assisting management and decision-making.

**Methods:**

ML models were developed using data of 404 ESG procedures performed at 12 centers across Europe. Collected data included clinical and demographic variables at the time of ESG and at follow-up. Multicentre/external and single center/internal and temporal validation were performed. Training and evaluation of the models were performed on Python’s scikit-learn library. Performance of models was quantified as receiver operator curve (ROC-AUC), sensitivity, specificity, and calibration plots.

**Results:**

Multicenter external validation: ML models using preoperative data show poor performance. Best performances were reached by linear regression (LR) and support vector machine models for TWL% and EWL%, respectively, (ROC-AUC: TWL% 0.87, EWL% 0.86) with the addition of 6-month follow-up data.

Single-center internal validation: Preoperative data only ML models show suboptimal performance. Early, i.e., 3-month follow-up data addition lead to ROC-AUC of 0.79 (random forest classifiers model) and 0.81 (LR models) for TWL% and EWL% achievement prediction, respectively. Single-center temporal validation shows similar results.

**Conclusions:**

Although preoperative data only may not be sufficient for accurate postoperative predictions, the ability of ML models to adapt and evolve with the patients changes could assist in providing an effective and personalized postoperative care. ML models predictive capacity improvement with follow-up data is encouraging and may become a valuable support in patient management and decision-making.

**Supplementary Information:**

The online version contains supplementary material available at 10.1007/s00464-023-10520-0.

Endoscopic sleeve gastroplasty (ESG) is a relatively new bariatric endoscopic procedure, which has proven to be safe and effective to reduce weight and obesity-related comorbidities [1–4]. ESG is scarless, repeatable, and reversible [5]. Thus, ESG has the potential to reach a larger proportion of obese patients compared to bariatric surgery [1], including those unfit for surgery, and those not undergoing surgery out of fear of invasive procedures [6]. Short- to mid-term results are promising [1, 7–9]. However, current ESG indications, as well as the endoscopic technique, have just started to be standardized and defined [10]. Although ESG predictors of success have been investigated in multiple studies [11, 12], the heterogeneity of data may represent an obstacle for thorough analysis with classical statistical methods. The increasing amount of diverse data gathered daily in endoscopic practice offers greater potential for standardized, data-driven approaches as machine learning (ML) [13]. ML models have been developed previously to predict complications and outcomes in other more invasive bariatric procedures [14], but never before for ESG. This study’s primary objective is to develop and evaluate ML models to predict ESG outcomes. The study is multicentric, featuring both temporal and external validation [15] to investigate generalizability of the models. Additionally, the proposed ML models can automatically adapt and evolve becoming more accurate with additional data during patient follow-up.

## Methods

### Dataset overview and outcome variables

Between 2016 and 2021, a database of 938 ESG procedures performed at 12 centers across Europe was established. Data were collected using a secure decentralized online registry by Medrio (Medrio inc. San Francisco, CA, USA). Longitudinal data at ESG follow-ups (months 1, 6, and 12) were collected retrospectively for procedures between 2016 and 2018, and prospectively after 2018. This study included all patients aged older than 18 undergoing ESG as the primary bariatric endoscopic/surgical procedure. Institutional Review Board (IRB) approval was obtained in each center and provided prior to accessing the online registry. Collected data shown in Table 1 of Supplements included demographic and clinical preoperative data such as blood tests and the presence of weight-related diseases. The outcome variables predicted by the ML models were total body weight loss (TWL) > 10% and excess body weight loss (EWL) > 25% at 12 months after ESG [16].

**Table 1:** Cohort description

Validation (no of cases)	Dataset (no of cases)	Follow-up month	BMI	TWL%	EWL%	DIABETES	HYPERTENSION
Multicenter external validation (270)	Training (136)	M0	38.1 (STD 4.9)	NA	NA	NA	NA
		M6	32.8 (STD 4.4)	14.5 (STD 6.6)	45.6 (STD 24.5)	NA	NA
		M12	32.3 (STD 5.4)	15.0 (STD 9.5)	48 (STD 14.7)	NA	NA
	Testing also Single center internal validation dataset (134)	ESG	40.6 (STD 7.4)	NA	NA	34/134	49/134
		M6	36.2 (STD 7.3)	14.5 (STD 8.1)	38 (STD 22.3)	NA	NA
		M12	36.7 (STD 8.3)	13.8 (STD 9.8)	35.8 (STD 26.3)	24/134	39/134
Single-center temporal validation (134)	Training (81)	M0	41.5 (STD 8.4)	NA	NA	26/134	39/134
		M6	37.2 (STD 8.2)	14.3 (STD 7.5)	36.5 (STD 19.9)	NA	NA
		M12	38.1 (STD 9.3)	12.9 (STD 8.1)	32.9 (STD 21.2)	19/134	30/134
	Testing (53)	ESG	39.3 (STD 5.4)	NA	NA	8/134	17/134
		M6	34.5 (STD 5.2)	14.7 (STD 9.0)	40.4 (STD 25.5)	NA	NA
		M12	34.6 (STD 6.2)	15.0 (STD 12.0)	40.3 (STD 32.2)	5/134	9/134

Multicenter external validation ML model training and testing dataset including 270 cases of ESG performed at 12 centers across Europe. Single-center internal validation ML models including 134 cases of ESG performed at a single center (IHU-NHC Strasbourg, France)

### Model training

ML models were trained to predict outcomes using multicenter validation i.e., external validation, and also single-center validation i.e., internal and temporal validation.

Four ML models were compared in this study: linear regression (LR), K-nearest neighbors (KNN), support vector machine (SVM), and random forest classifiers (RFC). They were selected based on their successful application in related tasks such as abdominal surgery outcome prediction [17]. All models were trained first using only preoperative clinical features, and subsequently trained with increasing amounts of follow-up variables (at 3-, 6-, and 9-month follow-up time points). This was performed to test our hypothesis that additional follow-up data, available during the routine clinical course, provided greater accuracy in ML prediction.

Models were trained and evaluated using Python’s scikit-learn library (version 1.0.2). Default model parameters were used for KNN (*K* = 5, weights = uniform, metric = minkowski), SVM (kernel = rbf, degree = 3), LR (solver = lbfgs), and RFC (*n*_estimators = 100, criterion = gini). Each model was trained to predict whether the procedure would be successful at 12-month follow-up (a binary output), including confidence estimates where applicable (SVM and LR). Success was defined with respect to TWL (≥ 10%) and EWL (≥ 25%), since both TWL and EWL were relevant clinical outcomes in ESG [18, 19]. Consequently, each model was trained twice; first to predict TWL success, and secondly to predict EWL success.

Model performance was quantified using the following established metrics: sensitivity, specificity, area under the receiver operator curve (ROC-AUC), and calibration plots.

### Model validation

#### Multicenter validation

In the multicenter validation, 136 of 638 cases from centers 2 to 12 were used to train ML models. The models were then tested on 134 of 255 cases from center 1. No patient information from the center 1 was used to train the ML model in this validation. Consequently, this validation measured the performance of the ML models using patient data from a center completely unknown to the model during its development (also known as an external validation).

#### Single-center validation

In addition to the external validation, ML models were developed and tested using just the data collected in the 1st center. The motivation was twofold: first, to investigate performance when the ML was specifically adapted to data from a single center, which may result in a more accurate model, albeit one that could only be used at a specific center, and secondly, to investigate if the additional variables available in the 1st center resulted in better performance. Two forms of validation were performed in the single-center validation. Validation was conducted using RFE and tenfold cross-validation (known as an internal validation). To preserve the same class ratio in all folds, stratified tenfold cross-validation was used in the following manner. First, patients from center 1 were randomly assigned to 10 disjoint sets (‘folds’). The first fold was then taken, and data from patients in all other folds were used to train the ML models. The trained models were then tested in patients in the first fold. The process was repeated 10 times until all folds were used to test the models. Finally, model performance across all 10-folds was averaged and reported. A temporal validation was performed as follows. First, the procedures were sorted in time, and then data from the 81 earliest procedures (60% of patients) were used to train the ML models. Finally, data from the remaining 53 procedures were used to test model performance. Consequently, the temporal validation assessed the ability of the ML models to predict outcomes of future patients from the same center.

### Data preparation

Clinical variables (‘model features’) that were not relevant for outcome prediction were automatically detected and removed using Recursive Feature Elimination (RFE) [20]. This established technique initially considered all features as independent variables, then features with the least impact on model predictions were subsequently detected and removed (pruned) until a target number of features remained. The target number was determined automatically using cross-validation, implemented in software using the sklearn.feature_selection.RFE (version 1.0.2) Python package. For multicentre model development, only 136 of 683 cases collected in center 2 to 12 had outcome variables, i.e., 12-month follow-up data, and could be included in training and testing of the models. Single center models as well as temporal validation models were trained and tested on 134 of 255 cases collected in center 1 as a result of missing outcome data. Missing feature values, e.g., unreported weight at follow-up, were handled using a combination of feature rejection and imputation. Specifically, all features with fewer than 50% recorded values, representing highly unreported features, were automatically removed. Of the remaining features, missing records were automatically imputed [21] using the variable’s mean (continuous variables) and mode (categorical variables).

## Results

### Cohort statistics

Descriptive statistics for the study sample are presented in Table 1. Variables collected in the 1st center and the remaining centers are listed in Table 1 of Supplements.

### Multicenter external validation

**Fig. 1:**
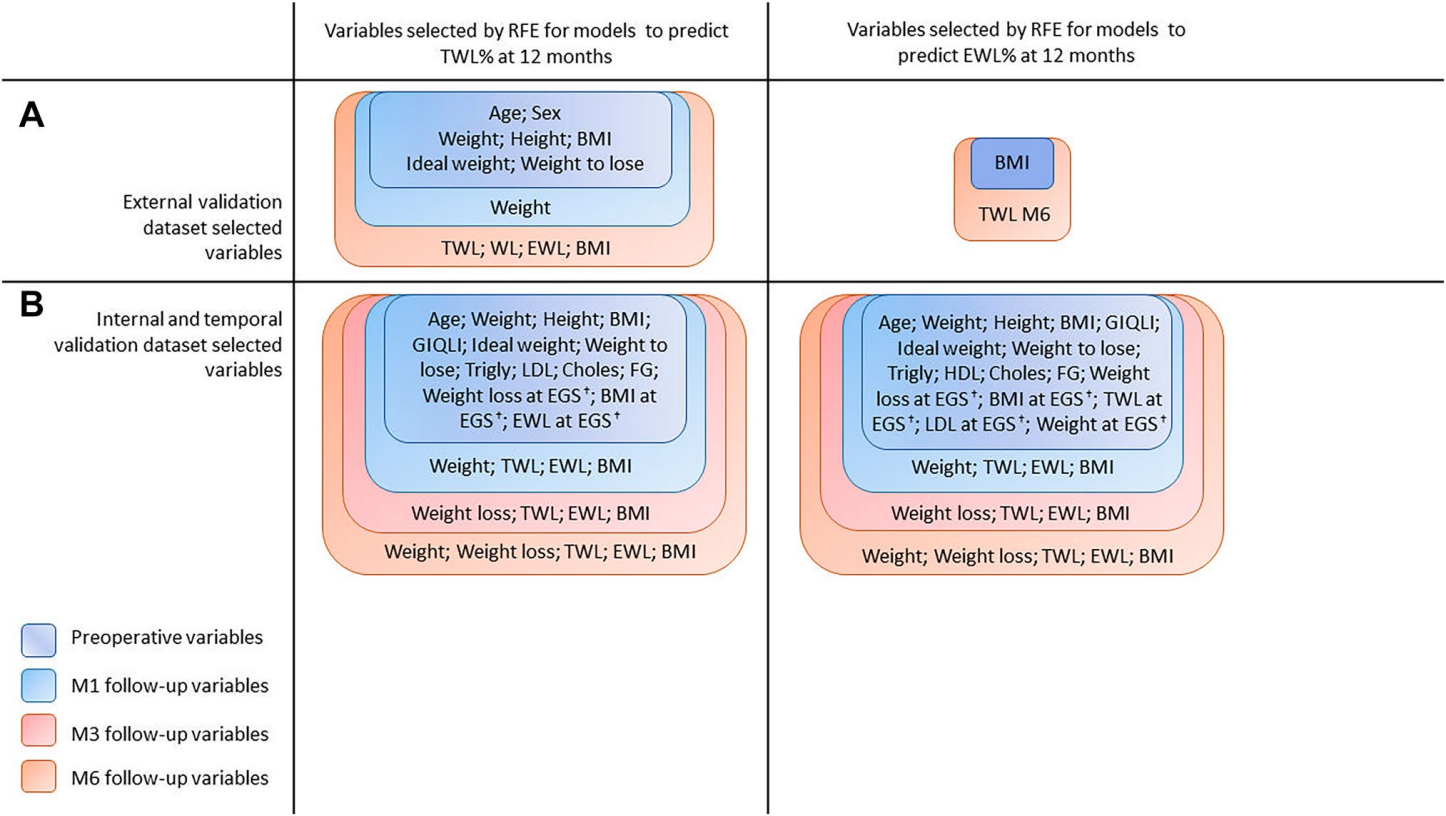
Recursive feature elimination (RFE) [32] was used combined with cross-validation (CV) for automatic relevant feature selection. To reduce overfitting, CV splits the dataset in subsets called k-folds, then machine learning models are iteratively trained on k-1 folds with the remaining fold serving as the test set. **A** Variables selected using recursive feature elimination (RFE) for prediction of TWL% and EWL% achievement in external validation models dataset. **B** Variables selected using recursive feature elimination (RFE) for prediction of TWL% and EWL% achievement in internal and temporal validation models dataset. *AHT* arterial hypertension, *BMI* body mass index, *choles* cholesterol, *ESG* endoscopic sleeve gastroplasty, *EWL* excess weight loss, *FG* fasting glucose, *GIQLI* Gastrointestinal Quality of Life Index, *LDL* low-density lipoprotein, *HDL* high-density lipoprotein, *trigly* triglycerides, *TWL* total weight loss

In the training dataset (136 patients from centers 2 to 12), mean age was 48.9 (SD 9.8) years, and 77.9% of patients were women. Total weight loss > 10% and EWL > 25% at 12 months were achieved in 68.4% and 76.5% of cases, respectively. In the test dataset (134 patients from center 1), mean age was 45.5 (SD 12.7) years, and 62.7% were women. Total weight loss > 10% and EWL > 25% at 12 months were achieved in 62.7% and 60.4% of patients, respectively. Clinical features that were selected automatically with RFE for predicting TWL and EWL success are shown in Fig. 1a.

**Fig. 2:**
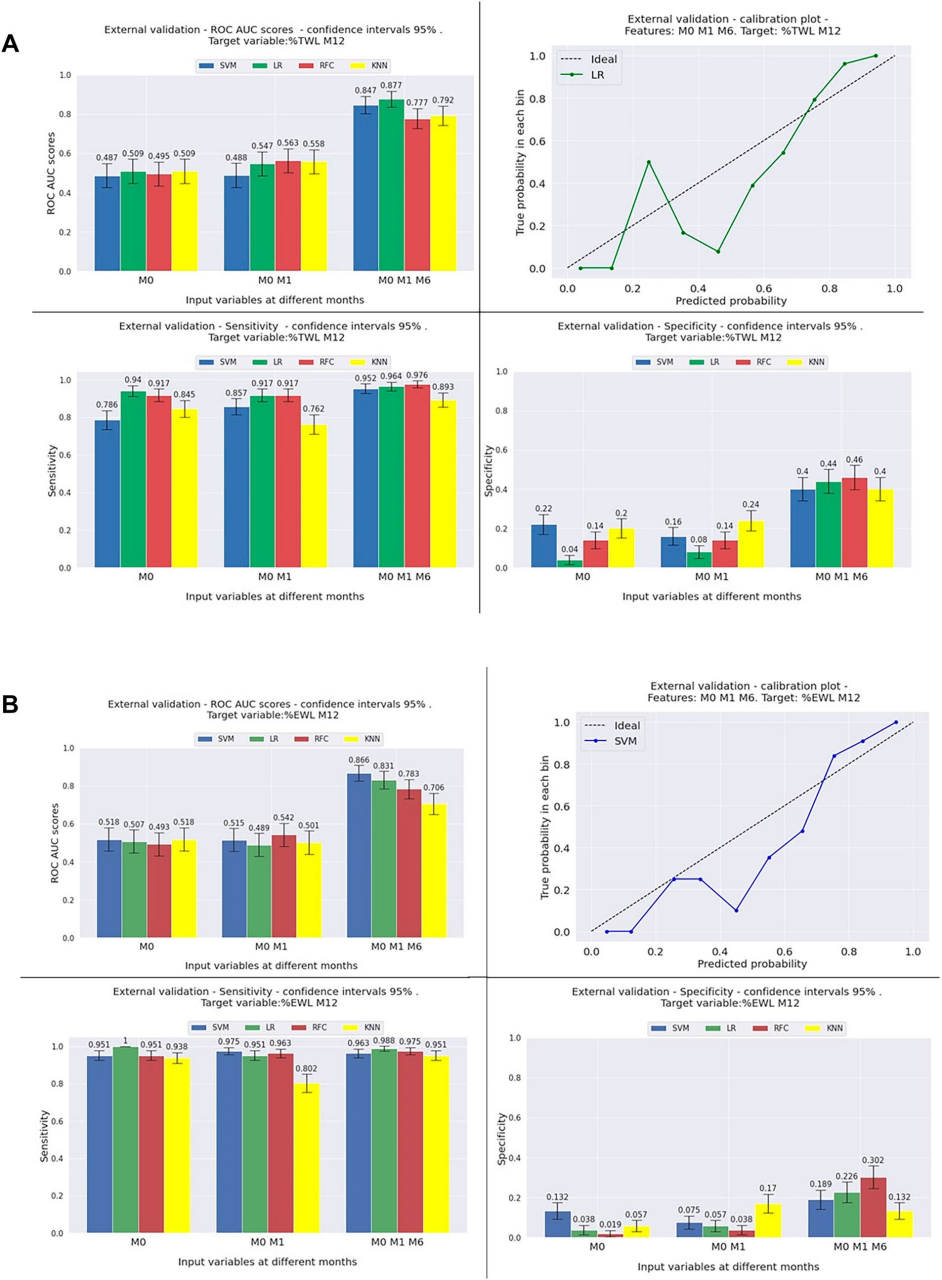
External validation results. Performances of multicenter external validation ML models expressed as ROC-AUC, sensitivity and specificity, and the calibration plot. The bar charts show the mean performance of the ML models, grouped into three time points: ‘M0’ represents performance of each ML model using only preoperative variables as inputs, ‘M0 M3’ represent performance using preoperative and 3-month follow-up variables. ‘M0 M3 M6’ represent performance using preoperative, 3-month and 6-month follow-up variables. Error bars represent 1 standard deviation/standard error. **A** TWL > %10 at 12 months ML models. **B** EWL > 25% at 12 months ML models. *EWL* excess weight loss, *KNN* K-nearest neighbour, *LR* linear regression, *M* months, *RFC* random forest classifier, *SVM* support vector machine, *TWL* total weight loss

Predictive performances for the multicenter external validation are shown in Fig. 2a for TWL% and Fig. 2b for EWL% prediction, expressed as ROC-AUC curves, sensitivity, specificity, and calibration plots. Models are designed to perform an initial prediction based on preoperative data. The models were then updated during follow-up.

### Single-center internal validation

**Fig. 3:**
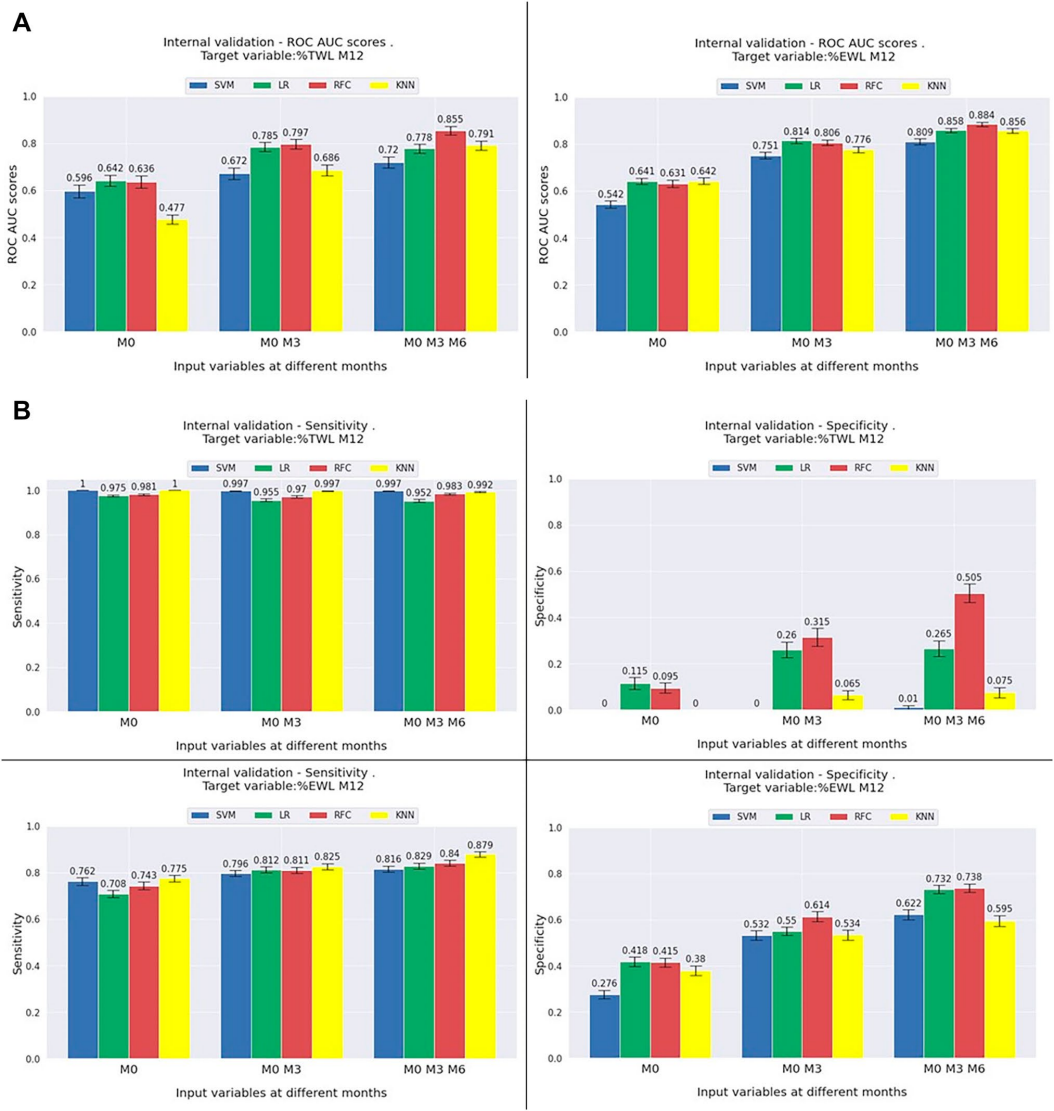
Internal validation results. Performances of single-center internal validation ML models expressed as ROC-AUC, sensitivity and specificity, and the calibration plot. The bar charts show the mean performance of the ML models, grouped into three time points: ‘M0’ represents performance of each ML model using only preoperative variables as inputs, ‘M0 M3’ represent performance using preoperative and 3-month follow-up variables. ‘M0 M3 M6’ represent performance using preoperative, 3-month, and 6-month follow-up variables. Error bars represent 1 standard deviation/standard error. **A** ROC-AUC for TWL > %10 and EWL > 25% at 12 months ML models. **B** Sensitivity and specificity of TWL > %10 and EWL > 25% at 12 months ML models. *EWL* excess weight loss, *KNN* K-nearest neighbour, *LR* linear regression, *M* months, *RFC* random forest classifier, *SVM* support vector machine, *TWL* total weight loss

Descriptive statistics are provided in Table 1. Clinical features that were selected automatically with RFE for predicting TWL and EWL success are shown in Fig. 1b. The ML model performances are shown in Fig. 3a using ROC-AUC for TWL% and EWL% and 3b showing models sensitivity, and specificity for TWL% and EWL% models.

### Single-center temporal validation

In the training dataset, 61.7% and 59.3% of cases reached TWL > 10% and EWL > 25% at 12 months, respectively. In the testing dataset, 64.2% and 62.3% of cases reached TWL > 10% and EWL > 25% at 12 months, respectively. Mean age was 47.8 (SD 11.8) and 42.1(SD 13.3) years in the training dataset (81 of 134 patients) and test dataset (53 of 134 patients), respectively. In the training dataset, 70.4% were women and 29.6% were men. In the test dataset, 75.5% were women and 24.5% were men.

**Fig. 4:**
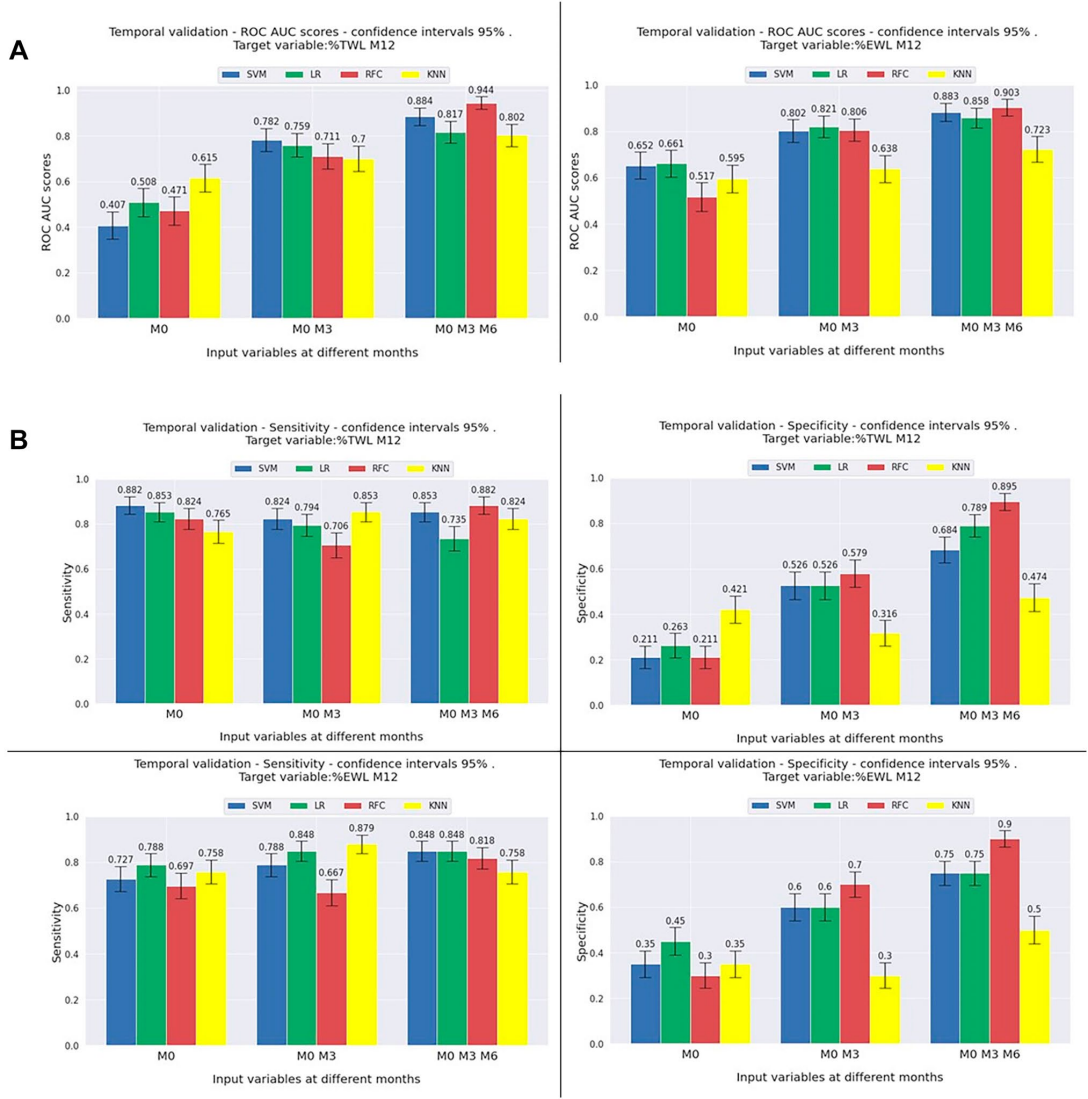
Temporal validation results. Performances of single-center temporal validation ML models expressed as ROC-AUC, sensitivity and specificity, and the calibration plot. The bar charts show the mean performance of the ML models, grouped into three time points: ‘M0’ represents performance of each ML model using only preoperative variables as inputs, ‘M0 M3’ represent performance using preoperative and 3-month follow-up variables. ‘M0 M3 M6’ represent performance using preoperative, 3-month, and 6-month follow-up variables. Error bars represent 1 standard deviation/standard error. **A** ROC-AUC for TWL > %10 and EWL > 25% at 12 months ML models. **B** Sensitivity and specificity of TWL > %10 and EWL > 25% at 12 months ML models. *EWL* excess weight loss, *KNN* K-nearest neighbor; *LR* linear regression, *M* months, *RFC* random forest classifier, *SVM* support vector machine; *TWL* total weight loss

In this validation, the ML models were trained only once as described above (model validation—single-center validation). The results are shown in Fig. 4a using ROC-AUC for TWL% and EWL% models, and 4b showing sensitivity, and specificity metrics for TWL% and EWL% models, respectively.

### Overall results

Multicenter external validation—predictive performances of the ML models using only preoperative data were poor, with the highest predictive capacity at 0.6 ROC-AUC in LR models. Predictive capacity increased when follow-up data were added, but better performance was achieved only when month 6 data were added to the ML models. This highlights the importance of short-term follow-up, which should guide clinical decision-making.

Single-center internal validation—ML models using preoperative data only show suboptimal ROC-AUC for both TWL% and EWL% achievement prediction. The addition of early, i.e., 3-month follow-up data, however, enables better predictive performance with ROC-AUC of 0.79 and 0.8 for TWL% and EWL% prediction, respectively. Adding data from 6-month follow-up further increases predictive capacity to ROC-AUC of 0.85 and 0.88 for TWL% and EWL% outcome prediction.

Single-center temporal validation—the temporally validated ML models show similar results to those internally validated, with suboptimal predictive capacity using only preoperative data, and better performance (ROC-AUC up to 0.8) with the addition of early, i.e., 3-month follow-up data.

## Discussion

This study aimed at developing and validating ML models to predict successful ESG outcomes across different centers. The exact place of ESG in the management of obesity remains somewhat uncertain. While various studies have been undertaken to examine patient selection for ESG [22] and its postoperative management, there is currently no standardized framework for defining success in terms of weight loss or assessing ESG impact on comorbidities and quality of life. In addition, there is still a lack of standardization of the ESG technique which results in a considerable variability in weight loss outcomes as indicated by a substantial standard deviation.

Patient data in this context are heterogeneous and continually evolving, emphasizing the need for data-driven approaches such as machine learning (ML) models to address these complexities.

ML predictive models are increasingly being developed and used in the medical field [23], showing good overall predictive outcomes [24], thanks to their ability to handle a large number of heterogeneous data and to identify patterns that would be missed by classic statistical models. Some studies have already presented models able to update predictions [25, 26], which is particularly crucial as patient characteristics regularly change and evolve. Models able to integrate preoperative, intraoperative, and postoperative information that accumulates throughout the patient pathway can provide objective data-driven support for clinical decision-making, as well as standardize and improve patient management. Some studies focused on identifying predictive factors of weight loss and comorbidity resolution after bariatric procedures [27–30], highlighting the interest in better patient stratification and care planning. Consistent follow-up by a multidisciplinary team and the patient’s compliance with therapeutic indications [11, 12, 31, 31, 32] have shown to be success predictors of ESG. Indeed, variables such as compliance with surgical, nutritional, and psychological follow-up visits and performance of physical therapy were collected in a subset of cases in center 1, and models developed on these data show good predictive performance with preoperative data (LR models with ROC AUC of 0.77 and 0.76 for TWL% and EWL% achievement at 12 months, respectively). However, these predictors are not easily measured quantitatively, making their role in clinical modeling impractical with standard approaches. In this study, preoperative and follow-up clinical variables easily collected in routine practice have been exploited using ML. The external validation of ML models exhibited poor results overall. That is, ML models trained on multicenter datasets and tested on data from an independent center could not be used to reliably predict outcomes at that center. This could be due to missing data and variations in procedure technique. Other possible influencing factors include population differences, ranging from cultural-behavioral to biological-genetic differences. Performances of ML models using temporal validation reached acceptable performances, but only when postoperative follow-up data were introduced. These findings indicated that ML models using only preoperative data may not be viably deployed in routine practice. The results are in line with prior studies addressing different surgical procedures, where the predictive performance of ML models, trained using only preoperative data, has been poor in general [23]. Preoperative data only may not be sufficient for accurate postoperative predictions. This could be understood by the fact that technical aspects of the intervention itself may influence the outcome, and that early to mid-term postoperative physiological changes in patients can lead to different outcomes. This study shows that ML predictive capacity improves by the integration of follow-up data, with a potential role in early postoperative patient management. ML models using 3-month follow-up showed strong results (AUC-ROC > 0.79 for TWL achievement at 12 months in the RFC models internal validation dataset), which is considered outstanding performance [33]. The internally and temporally validated ML models had higher predictive capacity than externally validated models, as was expected due to the higher similarity between training and testing samples. Internal validation normally represents an upper performance bound, so it clearly showed that accurate prediction of ESG outcome success using only preoperative data is likely impossible with ML. Among the developed ML models (external validation dataset), LR models show the best predictive performance, showing no advantage in more complex ML modeling processes, possibly facilitating their application. ESG indications and patient postoperative management include potential for resuturing or bridge to surgery. Although the safety and feasibility of performing laparoscopic sleeve gastrectomy or gastric bypass after ESG have been described [34, 35], the best timing as well as setting specific weight loss targets to transition to more extensive bariatric surgery have not yet been established. Predictive models could then help evaluate the best time to perform bariatric surgery after bridge ESG. Although primary ESG is effective, some patients may require revision procedures to augment weight loss [5]. Interestingly, information on resuturing after primary ESG may be integrated in the ML models to identify the best timing to perform the revision procedure. In addition, these cutting-edge ML predictive models could also be expanded to bariatric surgeries in which two-step procedures are proposed, such as single anastomosis duodenal switch (SADI-S) [36]. Our goal is to further enhance the ML model by integrating intraoperative video data and harnessing deep learning techniques. This will enable us to leverage the information embedded in videos, which can provide valuable insights into patients specific anatomy and variations in operator technique and skills.

### Study limitations

This study has some limitations: the relatively small size of the dataset compared to similar ML models development studies [17] may limit model performance. In addition, the relatively small dataset associated with a high rate of success in terms of TWL and EWL achieved leads to a very low sensitivity of ML models. Missing data, as well as lack of univocal definitions of some variables, e.g., the presence of comorbidities, also contribute to this study’s limitations. Indeed, models to predict obesity-related comorbidities were attempted, but lack of data prevented from developing reliable models. Many of the listed limitations highlight the importance of multicenter active collaboration and follow-up process standardization.

## Conclusion

To the best of our knowledge, this is the first study to develop ML models that predict the success of ESG with routinely collected clinical information. The ML model predictions are updated by the addition of follow-up data, adapting to changes in patients after ESG, improving outcome prediction and therapeutic strategies. In clinical practice, the use of common and easily collected variables should favor ML predictive models spread. Finally, the clinical usefulness of applying the ML models developed in this study should be investigated in impact phase trials.

### Supplementary Information

Below is the link to the electronic supplementary material.

Supplementary file1 (DOCX 13 kb)
