# Supplementary material for: Machine learning models to predict success of endoscopic sleeve gastroplasty using total and excess weight loss percent achievement: a multicentre study
Source: Surg Endosc. 2023 Nov 16;38(1):229–39. doi: 10.1007/s00464-023-10520-0 (PMC10776503; doi:10.1007/s00464-023-10520-0)
Supplement: Supplementary file 1 — Supplementary file1 (DOCX 13 kb) [file 464_2023_10520_MOESM1_ESM.docx]

| **MULTICENTRE COLLECTED VARIABLES** | **SINGLE CENTRE (centre 1) COLLECTED VARIABLES** |
| --- | --- |
| Patients enrolment in study | Patients enrolment in study |
| Age, Sex, Weight, Height, BMI, Diabetes, ATH, Systolic and Diastolic BP, GERD, Triglycerides, Cholesterol, HDL, LDL, HbA1c, Fasting blood sugar, Use of Hypertension and Diabetes drugs | Age, Sex, Weight, Height, BMI, ideal weight, weight to lose, ATH, GERD, Diabetes, OSAS, Triglycerides, Cholesterol, HDL, LDL, HbA1c, Fasting blood sugar, GIQLI |
|  | At procedure |
|  | Weight, BMI, TWL%, EWL% |
| M1 | M1 |
| Weight, Height, BMI, TWL%, EWL%, Diabetes, ATH, Systolic and Diastolic BP, GERD, Triglycerides, Cholesterol, HDL, LDL, HbA1c, Fasting blood sugar, Use of Hypertension and Diabetes drugs | Weight, BMI, TWL%, EWL% |
|  | M3 |
|  | Weight, BMI, TWL%, EWL% |
| M6 | M6 |
| Weight, Height, BMI, TWL%, EWL%, Diabetes, ATH, Systolic and Diastolic BP, GERD, Triglycerides, Cholesterol, HDL, LDL, HbA1c, Fasting blood sugar, Use of Hypertension and Diabetes drugs | Weight, BMI, TWL%, EWL%, ATH, GERD, Diabetes, OSAS, Triglycerides, Cholesterol, HDL, LDL, HbA1c, Fasting blood sugar, GIQLI, compliance to multidisciplinary team follow-up |
|  | M9 |
|  | Weight, BMI, TWL%, EWL% |

**Supplemental material**

**Table 1. Clinical variables collected in the multicenter registry and at the single center i.e. center No. 1. In the multicentre dataset, variables were collected at study enrolment and at 1, 3 and 6 month follow-ups. In center n°1, variables were collected at study enrolment as well as at procedure ^†^, 1, 3, 6 and 9 months follow-up.**

AHT, arterial hypertension; BMI, body mass index; EWL, excess weight loss; FG, Fasting Glucose; GERD, gastroesophageal reflux disease; GIQLI, Gastrointestinal Quality of Life Index; HDL, high density lipoprotein; LDL, low-density lipoprotein; OSAS, obstructive sleep apnea syndrome
